# Leveraging the Potential of Digital Technology for Better Individualized Treatment of Parkinson's Disease

**DOI:** 10.3389/fneur.2022.788427

**Published:** 2022-02-28

**Authors:** Holger Fröhlich, Noémi Bontridder, Dijana Petrovska-Delacréta, Enrico Glaab, Felix Kluge, Mounim El Yacoubi, Mayca Marín Valero, Jean-Christophe Corvol, Bjoern Eskofier, Jean-Marc Van Gyseghem, Stepháne Lehericy, Jürgen Winkler, Jochen Klucken

**Affiliations:** ^1^Department of Bioinformatics, Fraunhofer Institute for Algorithms and Scientific Computing, Sankt Augustin, Germany; ^2^Bonn-Aachen International Center for IT (b-it), University of Bonn, Bonn, Germany; ^3^Centre de Recherches Information, Droit et Societe, University of Namur, Namur, Belgium; ^4^Institut Polytechnique de Paris, Telecom SudParis, Evry, France; ^5^Luxembourg Center for Systems Medicine, University of Luxembourg, Esch, Luxembourg; ^6^Department of Artificial Intelligence in Biomedical Engineering, University of Erlangen Nuremberg, Erlangen, Germany; ^7^Asociacion Parkinson Madrid, Madrid, Spain; ^8^Institut du Cerveau (ICM), Paris, France; ^9^Department of Neurology, University Hospital Erlangen, Erlangen, Germany

**Keywords:** digital biomarker, Artificial Intelligence, precision medicine, digital health, Parkinson's Disease

## Abstract

Recent years have witnessed a strongly increasing interest in digital technology within medicine (sensor devices, specific smartphone apps) and specifically also neurology. Quantitative measures derived from digital technology could provide Digital Biomarkers (DMs) enabling a quantitative and continuous monitoring of disease symptoms, also outside clinics. This includes the possibility to continuously and sensitively monitor the response to treatment, hence opening the opportunity to adapt medication pathways quickly. In addition, DMs may in the future allow early diagnosis, stratification of patient subgroups and prediction of clinical outcomes. Thus, DMs could complement or in certain cases even replace classical examiner-based outcome measures and molecular biomarkers measured in cerebral spinal fluid, blood, urine, saliva, or other body liquids. Altogether, DMs could play a prominent role in the emerging field of precision medicine. However, realizing this vision requires dedicated research. First, advanced data analytical methods need to be developed and applied, which extract candidate DMs from raw signals. Second, these candidate DMs need to be validated by (a) showing their correlation to established clinical outcome measures, and (b) demonstrating their diagnostic and/or prognostic value compared to established biomarkers. These points again require the use of advanced data analytical methods, including machine learning. In addition, the arising ethical, legal and social questions associated with the collection and processing of sensitive patient data and the use of machine learning methods to analyze these data for better individualized treatment of the disease, must be considered thoroughly. Using Parkinson's Disease (PD) as a prime example of a complex multifactorial disorder, the purpose of this article is to critically review the current state of research regarding the use of DMs, discuss open challenges and highlight emerging new directions.

## Introduction

Digital technologies including wearable sensors, smartphone applications and smart algorithms currently receive a strongly increasing interest and begin to move toward medical applications. They have the potential to bring relevant information (e.g., “data”) derived from the patient's real-world environment to a better understanding of the diseases-related characteristics (“research”) and at the same time they potentially could serve for a better healthcare by supporting individual patients and doctors or other healthcare service providers. Digital biomarkers (DMs) derived from these digital technologies are specifically addressing the area of objective diagnosis and continuous real-world symptom monitoring. As such, a DM can be defined as an information—also referred to as “parameter,” “measure” or technically speaking as “data”—that is assessed or measured by digital technologies. Thus, DMs exhibit two major characteristics: First, they are measured by an objective method, e.g., passively *via* a wearable sensor, or actively *via* a questionnaire that is provided to the patient by a computer or smartphone app. This contrasts with any examiner-based questionnaire or examination procedure, which largely depends on the examiner's expertise and individual training. It is important to note, that the information itself could still be subjective which is especially the case for patient-reported outcome or experience measures (PROMs/PREMs), but the recording is objective.

A second characteristic of DMs is their patient centricity, which make them an ideal outcome revealing a patient's real-world environment, everyday procedures and activities of daily living outside regular physician visits (e.g., “real world data”). Here, it is important to note that DMs could also be derived from gold standard measures under routine procedures, including clinical examinations. The common aspect is, that they are derived from procedures that have not the intention to result into research data, but to improve healthcare, manage the disease or to measure everyday life activities of the patient on a continuous time scale.

**Figure 1:**
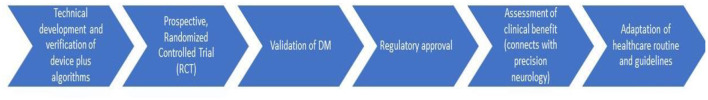
Stagewise process from development of Digital Biomarkers until their integration into routine healthcare.

Development of digital tools for this purpose is a stagewise process, where the last stage is the integration into standard healthcare (Figure 1):

1. A technical device measuring a certain signal needs to be developed. The signal needs to be analyzed *via* data analytical algorithms to extract a set of quantitative, informative features, e.g., gait parameters. These parameters can be understood as candidate DMs.
2. Using the device and corresponding algorithms, candidate DMs should be measured systematically within a randomized, controlled clinical study in parallel to traditional examiner-based outcome measures, e.g., Unified Parkinson's Disease Rating Scale (UPDRS).
3. The quantitative relationship of DMs to traditionally used outcome measures needs to be validated statistically, hence demonstrating that the new technique can be used as a valid diagnostic tool.
4. The technical device together with the employed algorithms for feature extraction needs to be approved by a regulatory agency.
5. The actual benefit of DMs compared to traditional outcomes with respect to an earlier or more accurate diagnosis or prognosis for the individual patient should be evaluated. Notably, this aim connects DMs with the broader ambitions of precision medicine, as outlined later.
6. Based on the outcome of step 5, existing guidelines and procedures for routine healthcare need to be adapted or newly implemented.

It is important to understand that the process through steps 1–6 is an interdisciplinary effort, which requires expertise from clinicians, engineers, statisticians and computer/data scientists. In addition, there are ethical, legal and social implications, which need to be addressed appropriately, specifically within step 6. Using Parkinson's Disease (PD) as a prime example of a complex multifactorial disorder, the purpose of this article is to critically review the current stage of research regarding the use of DMs and to highlight its interdisciplinary character. Moreover, we want to point out the connection with the broader ambitions of precision neurology as an emerging topic.

## Current State of Digital Biomarkers in Parkinson's Disease

### The Need for Early and Accurate Diagnosis of Parkinson's Disease

PD is a long-term neurodegenerative disorder. Parkinson's Disease is estimated to currently affect 7–10 million people worldwide (1). This places PD after Alzheimer's disease, but with a faster growth. Its prevalence in industrialized countries is around 0.3% and increases with age: 1% of people over the age of 60 and up to 4% of those over 80 are affected (2). The prevalence of PD has doubled between 1990 and 2016, which may be explained by the rise in life expectancy, better diagnoses and environmental factors.

PD is characterized by progressive loss of dopaminergic neurons in the substantia nigra (located in the midbrain). This loss already begins in the prodromal stage of the diseases, but first motor symptoms appear after 30–60% of the dopaminergic neurons are lost, and 60–80% of their striatal endings have degenerated (3). Standard diagnosis is mainly based on clinical examination. For example, according to the UK Parkinson's Disease Society Brain Bank Diagnostic criteria PD is diagnosed, if bradykinesia and at least one of the following criteria are fulfilled: muscular rigidity, 4–6 Hz rest tremor, postural instability not caused by primary visual, vestibular, cerebellar or proprioceptive dysfunction.

The accurate and timely diagnosis of PD is critical for the success of symptomatic treatment.

Among motor symptoms, impairments of speech and voice often appear as early symptoms. Hence, a lot of research in the field of DMs has focused on the detection of PD by analyzing biometric signals of speech, voice, gait and handwriting. In the following we will discuss the existing work related to DMs in more detail.

### Digital Biomarkers for Speech and Voice Impairments

There is emerging evidence that voice dysfunction is an early sign of motor impairment in PD (4–6). According symptoms are known as hypokinetic disartria, resulting in reduction of prosody, irregularities in phonation and difficulties in articulation. So far, most authors have focused on detecting PD in a moderate to advanced stages of the disease from speech and voice data (7–13). In earlier research DMs have been extracted as handcrafted features, such as e.g., repeated vowels or repeated sentences. According information was either obtained by asking subjects to read a text, by analysis of free speech, or by executing some more specific task, e.g., diadochokinetic (DDK) tasks (14). The DDK tasks consist of producing rapid syllabic sequences (/pa/-/ta/-/ka/, /ba/-/da/-/ga/, …) containing consonant-vowel combinations. They can reveal articulatory problems in the coordination of the motor subsystems involved in speech production. A more comprehensive review of existing methods for handcrafted feature engineering for PD detection is provided in (15).

Recent research shows that it is also possible to automatically discriminate PD from healthy controls at an early stage *via* handcrafted features extracted from speech (16). Moreover, in (17) a new DM technique (named X-vectors) was presented, which employs modern machine learning (ML) methods rather than designing handcrafted features. Improved classification results with X-vectors were obtained, validating the approach for early PD detection, using both high-quality speech recordings performed in laboratory settings and speech data acquired through telephone recordings. Such validations are important to move from the in-the-hospital controlled environment to remote eHealth applications.

### Digital Biomarkers for Hypomimia

In addition to voice and speech impairments, hypomimia (reduced facial dynamics, also known as facial bradykinesia) is a prominent clinical feature in people with PD and seen as secondary sign of the disease in an early stage (18). Bologna et al. summarize the main clinical and pathophysiological features of facial bradykinesia in PD (19): Clinical observations suggests that reduced spontaneous and emotional facial expressions are features of facial bradykinesia in PD. In PD, facial bradykinesia is primarily mediated by basal ganglia dysfunction leading to abnormalities of spontaneous, emotional and voluntary facial movements. Given associations between facial movement and voice, the potential of the Lee Silverman Voice Treatment (LSVT) to alleviate decreased facial expressivity was examined in (20).

Surprisingly, there are only few studies that tried to extract DMs for quantifying hypomimia: Vinokurov et al. extracted hypomimia DMs in Parkinson patients using a depth camera (21). Bandini et al. analyzed facial expressions in PD through video-based automatic methods (22). Grammatikoupoulou et al. developed PD detection tests, based on the interaction of users with everyday technological devices to quantify the progressive decrease of variability of facial expressions in early PD patients by analyzing patterns emerging from selfie-photos (23). Rajnoha et al. proposed algorithms to detect hypomimia based on face recognition methods (24). Jin et al. diagnosed Parkinson's disease *via* facial expression recognition from video analysis (25). In those studies algorithms from traditional machine learning and advanced deep learning were utilized.

Recently, deep learning methods have dramatically improved the state-of-the-art performance of face recognition, leaving space for extracting new types of hypomimia associated DMs (26). In addition, easy-to-use high-quality cameras are now more and more present in our living spaces. Therefore, there is potential to extract higher quality DMs related to hypomimia. A further and largely unexplored research direction is the combination of simultaneously acquired DMs from face and speech.

### Digital Biomarkers for Gait Impairment

Among other motor symptoms, PD results in walking impairments, which characterize disease progression, but also severely limit quality of life, increase fall risk, and leads to a great burden of patients (27, 28). Available gait assessment modalities include muscular activity, kinetic data (via force plates or pressure sensors), and kinematic information (via conventional optical motion capture systems or wearable inertial sensors), which can be used for PD severity analysis (29). Compared to gait pathologies arising in other diseases, most research has been published related to PD (29). Systematic changes in parameters such as gait speed, stride length, cadence, double support time have been identified in a large body of evidence (30). Gait parameters assessed by wearable digital technology, such as inertial measurement units which are worn on body segment such as foot, pelvis, lower back, or wrist, have been suggested to be used as digital biomarkers (31).

A recent systematic review summarizes current evidence on discriminant ability, construct validity, prognostic value and responsiveness using those digital mobility outcomes in PD (32). Recent research aims toward assessing gait in real-world walking environments, overcoming the challenges of standardized tests in clinics, which are also performed only rarely in a patient's disease history (33, 34). Current challenges include the technical and clinical validation, as well as regulatory acceptance of wearable devices for mobility assessment (35).

However, PD progression trajectories are heterogeneous and require a better understanding by modeling large data sets. Intra- and inter-individual variability and medication effects need to be accounted for when modeling disease progression longitudinally (36). Besides the classification of PD disease states, analytical approaches also include symptom detection. A recent work presented the detection of PD motor states at a high temporal minute-wise resolution (37). Notably, gait-based DM can also be combined with markers derived from other modalities to provide more reliable diagnosis, real-world symptom monitoring and prognosis. However, the availability of sufficiently labeled clinical reference datasets of adequate sample size currently remains a challenge.

### Digital Biomarkers for Impairment of Handwriting

Several studies have shown the potential of PD assessment through handwriting analysis, especially online handwriting acquired from tablet computers. Corresponding data provides rich information about spatio-temporal dynamics, such as velocity, acceleration, jerk (the derivative of the acceleration), and pressure of the handwriting strokes (38–40). To acquire the data, different tasks are usually proposed to PD patients and healthy controls, chiefly among them, writing words and sentences, copying names and addresses, drawings like Archimedean spirals, circles, and “llll” series. These studies, especially those based on statistical analysis tools show that, even at an early stage, Parkinsonian handwriting can get degraded in several ways, such as decrease of the stroke size, i.e., micrographia (41, 42), onset/increase of tremor (increase of jerk) (43–45), bradykinesia (slowness of movement) (41, 46, 47), and rigidity (48, 49).

These studies provide insights into the principal feasibility of DMs to capture differences between PD patients and healthy controls. However, the use of digital handwriting analysis in clinical routine requires to not only measure different features of the impairment, but also to aggregate these features into an overall diagnosis/clinical score. Only few authors have performed research in this direction so far: In (50), for instance, based on kinematic and pressure data, several ML classifiers have shown promising performance for discriminating PD against healthy controls. More recently, following the large success of neural networks in computer vision and speech analysis, several deep learning-based approaches have been proposed for PD assessment (51–53).

Despite these promising developments there are still several limitations in current research. First, current studies do not always target early stage PD. In addition, virtually all published approaches do not include the isolated rapid eye sleep movement disorder (iRBD) group, which limits their aid-to-diagnostic potential. Moreover, each group (PD or healthy control) is usually assumed to feature a unimodal handwriting behavior, which does not usually reflect the actual dynamics of each group, associated with different factors such as age, clinical scores (e.g., MDS-UPDRS, education, emotional states, etc.).

### Digital Biomarkers Derived From Other Biometric Data Modalities

There are also some recent studies analyzing biometric data modalities other than speech, face, gait and handwriting in order to extract DMs for PD. Giancardo et al. studied computer keyboard interaction as an indicator of early PD disease (54). Williams et al. used standard smartphone video recordings of finger tapping for the same purpose (55). Iakovakis et al. estimated motor impairments *via* touchscreen typing dynamics toward PD detection from data harvested in-the-wild (56). The assessment of the cardinal symptom tremor is addressed in several research studies as well (57–59). De Oliveira et al. reported early diagnosis of Parkinson's disease using EEG, ML and partial directed coherence (60). Their goal was to classify participants into three distinct groups: PD patients who are medicated; patients with PD and drug deprivation; and healthy subjects. Even though their results are promising, they require specific sensors and are therefore not suited for remote monitoring. In addition, extracted DMs have not been tested and validated in different cohorts. In conclusion, DMs extracted from data modalities other than speech, hypomimia, gait and handwriting could potentially give new insights into the pathophysiology of PD.

### Validation and Regulatory Approval of Digital Biomarkers

Before any use in clinical practice, a candidate DM needs to be validated *via* a prospective clinical study, like any molecular biomarker. The study needs to show that the DM is applicable to the target population in the context of its intended use (61). That means the correlation of the DM with disease state and/or any accepted clinical endpoint, such as MDS-UPDRS, has to be statistically demonstrated. Based on the outcome of such a study, a DM based solution may subsequently seek for approval as “software as a medical device (SaMD)” by a regulatory authority. Noteworthy, according to the definition of the US Food and Drug Administration (FDA), SaMDs can perform a medical function without necessarily being part of a hardware medical device (62). Hence, ML algorithms are covered by this definition.

Examples of approved medical devices, of which extracted features can be regarded as validated DMs include Mobile GaitLab, a CE certified mobile gait sensor producing various gait parameters that have previously shown to reflect a physician's rating of PD, and to correlate with MDS-UPDRS and drug response (63–66). Also, wrist worn sensors have been CE certified to detect various motor-impairments in PD such as bradykinesia, tremor, or dyskinesia.

It should be noted that the CE label and the according EU device classification regulates DMs from a purely technical perspective and does not provide any evaluation of medical benefit. Indeed, the regulation of DMs is in the US and EU organizationally separated from that of drugs, and thus far the experience with DMs in the context of clinical trials is altogether limited. Therefore, there exist uncertainties for pharma companies, which seek for approval of a drug: While from a scientific point of view measuring a drug's efficacy *via* a technically certified DM might be viable alternative to a traditional examiner-based clinical assessment, the acceptance of such a digital endpoint by a regulatory authority is currently not totally clear. Regulatory agencies first want to evaluate the relationship of DMs to traditional clinical endpoints and understand their benefit for measuring drug efficacy and safety. Hence, at present DMs are not yet included as standard into clinical trials in the PD field.

## Data Science as Enabler of Digital Biomarkers

### Digital Biomarkers Are Features Extracted From Large Volumes of Data

The high resolution of digital sensors allows for an unprecedented high volume of precise data that can be leveraged for assessment of PD symptoms. For example, a recent camera for video streaming (Microsoft's Kinect v2) provides five video related data streams. Besides the color (1,920 × 1,080 at 30 Hz) and infrared (512 × 424 at 30 Hz) data streams, it provides depth images (512 × 424 at 30 Hz), body index images (512 × 424 at 30 Hz) and the skeleton information for every tracked person (25 joints at 30 Hz). Color images are provided with 4 bytes per pixel and depth images with 2 bytes per pixel resolution. In order to distinguish tracked persons, the camera software stores body index images, which take one byte per pixel. The joint positions are provided at a resolution of 4 bytes per coordinate (12 bytes per joint). Every frame contains a time stamped gait assessment (67).

To find patterns in the resulting huge volume of data, advanced data analytical methods are required, including modern approaches from Artificial Intelligence (AI). The result of these algorithms is a set of abstract (not necessarily human understandable) features describing, for example, gait or voice characteristics within an individual patient. If such features can be reproducibly associated to disease symptoms, they can be regarded as candidate DMs, which after a rigorous validation process might subsequently be approved for use in medical practice.

From a technical point of view, there are several approaches for feature extraction from digital sensor data: One possibility is to construct handcrafted features, reflecting, for example, spatiotemporal or kinematic aspects of voice (68), handwriting (69) or gait (70). On the other hand, neural networks and, in general, representation learning-based approaches can directly process raw data to generate high-level features implicitly (51, 69, 71, 72). Learning of high-level, implicit features is usually guided by a supervised prediction task, for example discriminating PD vs. healthy controls (51, 71).

In addition to machine learning approaches, biomechanical models have also been used for feature construction, specifically in gait analysis (73–75).

### Unavoidable Challenges From a Data Science Perspective

It is important to mention that the derivation of DMs from real-world data is associated with several challenges from a Data Science perspective. First, real-world data are always and unavoidably impaired by noise. Noise can result from the technical measurement process as well as from the natural biological variability of disease symptoms. Discriminating both sources of noise is still a major challenge in Data Science (76).

Another challenge is the limited amount of training data, which is typical in biomedical applications due to the high costs and regulatory issues compared to other application fields. In addition, statistical and AI methods assume that training data was sampled independently and identically distributed (i.i.d.) from the same underlying statistical population. This assumption is rarely fulfilled in biomedicine, e.g., due to selective recruitment of patients. While modern Data Science techniques like transfer learning might partially address this issue, their actual benefit has still to be evaluated carefully and more broadly. Therefore, recent initiatives focusing on generation of large scale real-world, such as the “All of Us” research program in the US, could play an increasingly important role in future PD research (77).

Finally, it is worthwhile to mention that supervised learning approaches require a manual and careful labeling of cases by medical experts, which can be time consuming and depends on a certain gold standard. Accordingly, the application of the device is then limited to the used gold standard. While unsupervised approaches are well-established in Data Science, their use is limited to specific tasks (e.g., clustering, representation learning), and the validation of detected patterns is comparably much more difficult than in case of supervised learning.

In conclusion, Data Scientist face unique challenges in biomedicine, which require a careful adaptation of existing methods or the development of new ones (78).

## The Emerging Future: Digital Biomarkers in Precision Neurology

### Precision Neurology Requires Multi-Modal Data and Multivariate Stratification Approaches From the Area of Data Science

DMs can be viewed as objective and quantifiable biomarkers in the same way as a protein abundance, a genetic variant, a neurological image or any other patient specific characteristic that can be used for individualized disease diagnosis, monitoring, prognosis, drug response prediction or stratification. The main difference to traditional biomarkers is the measurement process, which in the case of a DM is performed *via* a digital device or *via* a smartphone app. In contrast to traditional biomarkers, the portability of most DMs offers unprecedented mobility and continuity of the measurements, enabling a location-independent sensitive monitoring of patient phenotypes for long durations and with minimal interruptions.

In most disease areas, including neurology, single biomarkers are not sufficient to reliably diagnose and monitor a disease, prognose its progression, predict the response to a specific drug or to identify well-separated patient subgroups. This is because many neurological diseases (including PD) are complex and heterogeneous, involving high inter- and intra-individual variability over time and affecting a multitude of biological mechanisms. Different combinations of genetic and environmental factors may lead to different forms or sub-types of the disease, which may manifest themselves in a wide spectrum of highly diverse motor- and non-motor symptoms. This diversity cannot be captured by a single biomarker. Accordingly, comprehensive multifactorial biomarker signatures are needed.

**Figure 2:**
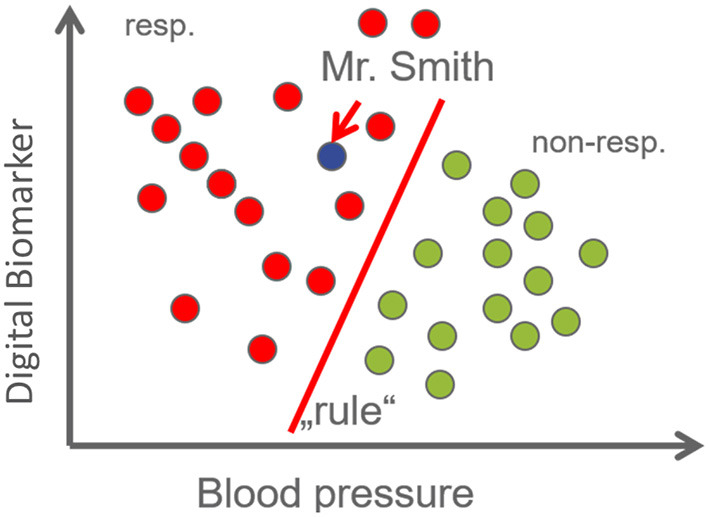
Machine learning (ML) refers to an approach, in which a statistical model is fit to data. After this “learning” process the model encompasses a “pattern” or a “rule.” ML can be supervised or unsupervised. In supervised learning, we train a model based on a dataset with hopefully many observations, each containing a (possibly large) number of features, coupled with the known clinical outcome (e.g., drug response, see example on the right). Based on the established model predictions for patients that were not part of the training data can be made (such as Mr. Smith). Machine learning models can make accurate predictions, even if there is no single biomarker that discriminates patient groups. For example, in the Figure neither blood pressure nor the digital biomarker alone allows for discriminating drug responders from non-responders. However, both features together admit a perfect separation (diagonal line). Notably, supervised learning is not restricted to classification, but also continuous outcomes can be predicted. As opposed to supervised learning, unsupervised learning aims at inferring patterns from data without having access to a label, such as phenotype. Many machine learning models can discard features that are irrelevant for the prediction (sparse models). The set of features selected by the algorithm then establishes a biomarker signature.

Identifying and robustly validating such marker signatures is difficult and requires state-of-the-art approaches offered by Data Science. Specifically, multivariate stratification and prediction algorithms using techniques from the area of Artificial Intelligence (AI, including machine learning) thus play an increasingly important role (Figure 2). Recent noteworthy examples from the area of PD include a machine learning approach to predict the risk of an individual patient to receive the clinical diagnosis PD using routinely collected data from electronic health records about 5 years in advance (79). A further example is a machine learning approach to cluster AD and PD patients into 4 different subgroups based on the genetic burden on 15 molecular mechanisms (80). The authors in (81) developed a machine learning approach to predict the progression of PD using a signature of 27 inflammatory cytokines measured in blood serum. Furthermore, mobile phone gyroscope and accelerometer data have been used in combination with demographic and clinical data to predict different measures of Parkinson's disease symptom severity (82). Finally, in (83) a subgrouping of PD patients based on their disease trajectories, described *via* a variety of outcome scores, was suggested.

Altogether, there is an increasing awareness that moving toward precision neurology requires a holistic view on the disease, in which aging, genetic and epigenetic variants, environmental pollutants/toxins, life style, comorbidities and clinical assessments are considered jointly (84–87). Accordingly, we believe that in the future DMs will play a more important role in this context, because they principally allow for a more objective, sensitive and continuous assessment of longitudinally variable disease symptoms compared to questionnaire-based outcome measures. Hence, DMs could in the future be combined with other data modalities (including genetic variants) for earlier, more robust and accurate disease diagnosis and prediction of disease progression.

In addition, DMs offer further opportunities: Certain disease comorbidities, such as freezing of gait in PD, which can be particularly debilitating for the patient, may require a continuous patient monitoring to enable new effective interventions. Such applications, necessitating uninterrupted monitoring and offering the potential to issue direct feedback and warnings to the patient, may only be achievable with the help of DMs.

Of course, the idea to use DMs for precision neurology requires a sufficient validation, and thus we initiated the dedicated EU-wide project DIGIPD (Validating DIGItal biomarkers for better personalized treatment of Parkinson's Disease—https://www.digipd.eu). DIGIPD will specifically focus on three types of DMs: (i) sensor-based gait assessment, (ii) voice recordings (also *via* phone), and (iii) video recordings of face movement. The project will initially evaluate the association of these DMs with disease diagnosis and progression, as described *via* traditional questionnaire-based assessments, such as MDS-UPDRS. Furthermore, we will test, in how far DMs could be used and interpreted in combination with other data types (genetics, demographics, molecular markers and associated cellular disease maps) to predict the progression of the disease for an individual patient, as well as other clinically relevant disease outcomes and comorbidities.

### Toward Establishing Precision Neurology in Clinical Practice

AI based stratification and prediction algorithms resulting from research projects such as DIGIPD are not immediately applicable in clinical practice. There is a multi-step validation procedure and legal pathway required before using any such approaches outside research (76): Typically, AI algorithms are initially validated internally by leaving out parts of the original training data for testing purposes, using a single split of the data into a training and a test set, or multiple random or stratified splits as part of a dedicated cross-validation procedure. This is necessary to evaluate how the algorithm can predict a given endpoint in principle, but it does not answer the question, in how far the algorithm might be applicable to patients that are different to those included into the original training cohort. This is important, because patient selection criteria and differences between patient populations in different geographic regions unavoidably bias studies used for training and testing AI algorithms. In consequence, the typically made assumption in machine learning of training and test data being drawn from the same underlying statistical distribution is certainly not fulfilled in medicine (88). Accordingly, subsequent validation steps for an AI algorithm include (76):

1. Retrospective validation on independent study data.
2. Prospective validation using a specifically designed clinical study
3. Regulatory approval as a medical product, as outlined above

In addition, the actual benefit for the patient compared to the current medical practice should be evaluated, also from a health economic perspective. Finally, since biomarker-guided treatment decisions may also involve adverse effects in some cases (e.g., in case of CSF biomarkers), not only a cost/benefit assessment may be required, but also a critical evaluation of the benefit/risk relation.

## Ethical, Legal, and Social Aspects

### The Use of Digital Biomarkers Requires Considering Ethical, Legal, and Social Aspects

In the recently published World Health Organization (WHO) global report on Artificial Intelligence in Health (89), Soumaya Swaminathan aptly emphasizes that while AI “has enormous potential for strengthening the delivery of health care and medicine […], for AI to have a beneficial impact on public health and medicine, ethical considerations and human rights must be placed at the center of the design, development, and deployment of AI technologies for health.” The collection of large volumes of health data through digital devices as well as the use of advanced analytical techniques to process these data entail multiple ethical, legal and social implications.

### Ensuring Ethical and Legal Data Processing

Collecting DMs for better individualized treatment of PD and sharing information pertaining to patients between different research centers raise questions regarding confidentiality of data, their cross-border flows (90), availability of data for research, and access to the results of the research. Because AI systems require large quantities of good-quality data for training and validation, Intellectual Property and data protection, are critical issues. Aikten et al. suggest that individuals are willing to support the use of medical data for technological development, but only if careful attention is paid to confidentiality, control, governance and assured use for public interest (91). This is complicated by the rapid movement of large technology companies (e.g., IBM, Google) into healthcare, as well as the proliferation of start-ups developing healthcare related products. Expansion of data use also increases opportunities for data leakage and re-identification (e.g., Google with the vast individuated and geolocated datasets they already hold), and this needs to be addressed to leverage the potential of digital technology in a justifiable and legitimate way (92).

The General Data Protection Regulation (GDPR) (93) imposes rules that may be difficult to reconcile with AI use, such as the principles of transparency, data minimization and access to the system underlying the decision taken by an AI system. Legal pathways are however possible, especially as scientific research is promoted by the Regulation (article 89.1) and national laws (article 89.2) by permitting and providing derogation measures for such aims. Furthermore, as protection of personal data is an “intermediate” right to protect fundamental values, namely the right to self-development/ autonomy/ human dignity, its rules are to be interpreted in this context (94).

Protection of the used technologies and databases against adversarial model attacks (95) is crucial as well. There is thus a need to identify the technical measures that need to be implemented to ensure resilience to attack and security of the systems in use. The GDPR addresses concerns relating to data security and confidentiality.

### Ensuring Ethical AI

The European strategic plan on AI released in 2018 (93) includes the provision of an ethical and legal framework for “Trustworthy AI.” In order to achieve this objective, the European Commission set up a High-Level Expert Group on AI, which published in 2019 the Ethics Guidelines for Trustworthy Artificial Intelligence (96). These Guidelines indicate that AI should be human-centric and used in the service of common good and humanity, with the aim to improve human welfare and freedom. Trustworthy AI must be lawful, ethical and robust, both from a technical and social perspective, since, even with good intentions, AI systems can cause unintentional harm. With respect to the ethical aspects, the Guidelines prescribe seven requirements:

- *Human agency and oversight*: AI systems should empower human beings, allowing them to make informed decisions and fostering their fundamental rights. At the same time, proper oversight mechanisms need to be ensured, which can be achieved through human-in-the-loop, human-on-the-loop, and human-in-command approaches.
- *Technical Robustness and safety*: AI systems need to be resilient, secure, reliable and reproductible, and provide accurate results.
- *Privacy and data governance*: full respect for privacy and data protection is to be ensured, with adequate data governance mechanisms.
- *Transparency*: the data, system and AI business models should be transparent. Traceability mechanisms can help achieving this. Moreover, AI systems and their decisions should be explained in a manner adapted to the stakeholder concerned.
- *Diversity, non-discrimination and fairness*: unfair bias should be avoided, while access to all should be ensured as well as multiple stakeholders' participation.
- *Societal and environmental wellbeing*: AI systems should be sustainable and environmentally friendly. Social and societal impacts should also be considered.
- *Accountability*: Auditability, report on negative impacts, and adequate redress mechanisms should be ensured.

The AI HLGE has developed a detailed list of measures to be adopted as well as the procedure to be followed in order to meet the seven requirements that must be covered by the so-called “ethical” evaluation: this is the “Assessment List for Trustworthy Artificial Intelligence” (ALTAI) (97). Given the specificity of AI system applications, it must be adapted to the particular case and context in which the system will operate. An online prototype has been developed to guide developers and deployers of AI systems through a dynamic and accessible checklist. While this list allows us to assess the conformity of the system envisaged with the above-mentioned ethical principles, it does not allow us to assess the conformity of the system with the legal requirements.

### Ensuring Human Oversight and Accountability

Some AI algorithms are based on ML, which is a fast, automatic and not an intuitively explanatory self-learning mechanism. ML algorithms are often described as transforming inputs to outputs through a “black box” which involves introducing a critical issue: *explainability* or *interpretability* of the output from a human perspective (98, 99). Many ML algorithms, specifically in the field of neural networks, produce outputs that are difficult for humans to explain or interpret. This is in contrast with traditional statistical modeling approaches (100). It could imply that future healthcare AI systems may recommend individualized diagnostic, prognostic and management decisions that lack transparency and thereby trust (101). This situation also challenges the requirement of transparency when it comes to the processing of personal data based on automated decision-making, as well as the data subject's right to information.

Tightly linked with the *explainability* and *interpretability* of the functioning of the system and its outputs, *responsibility* and *liability* are crucial issues regarding AI use (102). While AI is being introduced into healthcare exponentially, there is a lack of clear regulator, trial process and legal accountability regime (103). One exception is the area of data protection, especially protection of health-related data, where general and specific regimes are emerging or already adopted (including in the GDPR) and further work between stakeholders is undergoing. However, there is still a clear “regulatory gap” with respect to clinical use of AI. A new “purpose built” regulatory framework should be created, setting up guidelines and regulation to be followed protecting vulnerable patients but also ensuring that appropriate innovation in technologies is supported.

Furthermore, the use of AI in healthcare may lead to changes in the role and self-image of physicians. Future decision making is likely to involve both clinicians and AI systems in some way, requiring management of machine-human interaction. If physicians are directly involved in patient care, but if decisions partly depend on non-explainable AI recommendations, physicians will face challenges regarding their moral and legal responsibility. Hence, it should be considered where to locate the decision-points in algorithms' development that should trigger attributions of responsibility (104). This shift in attributions of responsibility may affect the patient's trust in clinicians and health care institutions and change medical roles. Clinicians could have more time to talk with patients, for example, but if decisions are algorithmically supported there may be limits to what clinicians are able to explain (105). It is thus important to ensure that technical means remain a help for the human. In addition, if algorithms enter individual clinical care and clinicians become more dependent on these systems for decision-making, patients who do not share the requested health data may not be able to receive gold-standard treatment, creating tension between clinical consent and quality of care (106). The eventuality of a right for patients to refuse the use of new techniques making use of AI systems in medicine has therefore to be considered.

### Avoiding Unfair Bias

The data feeding the AI system should be appropriate, accurate and up to date. The AI system's level of accuracy should be assessed, and this accuracy should be appropriately guaranteed. Fundamentally, AI systems are “made of” data. By exposure to massive datasets, they develop the ability to identify patterns in those datasets, and to reproduce desired outcomes. These abilities are shaped not only by their coding, but also by the data they are fed with. Feeding biased data into AI systems produces systematically biased outputs from those systems. In addition, human choices may prompt AI systems to work in discriminatory or exploitative ways (107). A high degree of transparency about data sources and distinction between efficacy and effectiveness will be necessary to protect against these potential weaknesses. Appropriate ways should be implemented to prevent, discover and correct mistakes.

### Ensuring Protection of Vulnerable People

Research with vulnerable people is an additional source of potential concerns. Vulnerable persons are described as those who are relatively (or absolutely) incapable of protecting their own interests (108) (p. 65). This includes, for instance, people with an incurable disease (as PD) or with physical frailty (e.g., due to age or co-morbidities). While research with this population is generally allowed unless a good scientific reason justifies their exclusion, there are some specific considerations that need to be addressed. According to the Declaration of Helsinki, “medical research with a vulnerable group is only justified if the research is responsive to the health needs or priorities of this group and the research cannot be carried out in a non-vulnerable group. In addition, this group should stand to benefit from the knowledge, practices or interventions that result from the research.” (109). This principle of providing specific protections and safeguards to vulnerable persons is reiterated in the UNESCO Declaration on Bioethics and Human Rights (art. 8) (110), the WHO Handbook for Good Clinical Research Practice (principle 1) (108) and the CIOSM Guidelines (guideline 15) (111). Such protections could include “allowing no more than minimal risks for procedures that offer no potential individual benefits for participants; supplementing the participant's agreement by the permission of family members, legal guardians, or other appropriate representatives; or requiring that the research be carried out only when it is targeted at conditions that affect these groups.”

### Ensuring Legally Trustworthy AI

While, as it was emphasized by the Committee on Social Affairs, Health and Sustainable Development of the Council of Europe (112), “the speed of the development and deployment of [AI] technological developments [in health care] is much faster than that of the legal framework regulating them,” any development of AI driven solutions for health care must be in line with existing legal instruments. These include the European Social Charter (113), the European Convention on Human Rights (114), the Convention on Human Rights and Biomedicine (115), the Convention for the Protection of Individuals with regard to Automatic Processing of Personal Data (116) and its Amending Protocol (117), and the European Charter on Medical Ethics (118).

In addition, AI systems may be considered as medical devices pursuant to the regulation on medical devices (119) which prescribes that “a device may be placed on the market or put into service only if it complies with this Regulation when duly supplied and properly installed, maintained and used in accordance with its intended purpose” (article 5). AI systems considered as medical devices and all elements permitting its functioning therefore need to be tested and controlled. It is also important to note that in April 2021, the European Commission proposed new rules on AI (120). The proposed regulation imposes multiple obligations on all actors involved in the development and use of AI systems qualified as high risks. According to Article 6 §1, the AI system shall be considered high-risk where it “is intended to be used as a safety component of a product, or is itself a product, covered by the Union harmonization legislation listed in Annex II; [AND] the product whose safety component is the AI system, or the AI system itself as a product, is required to undergo a third-party conformity assessment with a view to the placing it on the market or putting it into service of that product pursuant to the Union harmonization legislation listed in Annex II.” The regulation on medical devices already mentioned being listed in Annex II, the proposed AI Act may impose a multiple set of new obligations for AI use in the health sector.

### Ensuring Patients' Involvement

While DMs and associated AI algorithms may offer benefits for the individual patients from a scientific point of view, the patients' actual acceptance as well as their involvement in the development process is essential. This is strongly encouraged by UNESCO in its recently adopted Recommendation on the Ethics of AI (121). Hence, any attempt to implement DMs in healthcare routine should be accompanied by an engagement with patients. This engagement is necessary during all steps of the process: identification of research questions, study design, recruitment processes, data collection and analysis of results. It also seems necessary to provide some explanations regarding the apparent need to implement AI solutions that may transform the experience of healthcare provision and consumption, and to increase control and deliver investment opportunities (122).

As an example of such an engagement, within the EU wide project DIGIPD (https://www.digipd.eu), a structured online survey as well as interviews will be conducted to investigate the acceptance by PD patients of the use of sensitive personal data collected through digital devices and advanced analytical techniques in the development of better individualized patient care. Data privacy and ethical concerns will specifically be addressed. The primary objective of this approach is to collect detailed information on the opinions, thoughts, experiences and feelings of PD patients on the use of DMs (extracted from mobile gait sensors, voice recordings and face movements) and AI in clinical routine. A secondary objective is to identify other factors that must be taken into account in order to gain patients' acceptance and meet their demands.

## Conclusion

Recent years have witnessed a strongly increasing interest into DMs, because they enable a quantitative and continuous monitoring of disease symptoms within a patient's real-world environment. A main distinction point to traditional examiner-based outcome measures is thus the avoidance of a subjective rater bias. Furthermore, DMs can be regarded as non-invasive complements or even replacements of molecular biomarkers. In this context DMs could also play a vital role in the emerging field of precision neurology. However, research in this direction is just starting.

Current research demonstrates that DMs derived from speech, voice, gait, handwriting and face movement have a high potential in the PD field. However, their shift from research into medical routine requires a more systematic and rigorous validation within prospective clinical studies and subsequent regulatory approval. In addition, there are ethical, legal and social implications that need to be considered: DMs are in essence data driven and specifically AI driven techniques. Hence, appropriate data privacy measures need to be implemented and the trustworthiness of AI algorithms ensured. In addition, the implementation of DMs into healthcare routine requires acceptance by patients.

Altogether, the development of DMs and subsequent implementation into medical routine has to be regarded as a stagewise process, which requires input from clinicians, engineers, statisticians, computer/data scientists, legal experts, regulators and patient representatives. Accordingly, institutions that want to be active in this field need to be aware of the necessary organizational steps as early as possible.

## Author Contributions

JK: initiated the manuscript. HF: guided the project. HF, NB, DP-D, EG, FK, ME, MM, J-CC, BE, J-MV, SL, JW, and JK: drafted the manuscript. All authors contributed to the article and approved the submitted version.

## Funding

This project was partially funded by the ERA PerMed EU-wide project DIGIPD (01KU2110).

## Conflict of Interest

The authors declare that the research was conducted in the absence of any commercial or financial relationships that could be construed as a potential conflict of interest.

## Publisher's Note

All claims expressed in this article are solely those of the authors and do not necessarily represent those of their affiliated organizations, or those of the publisher, the editors and the reviewers. Any product that may be evaluated in this article, or claim that may be made by its manufacturer, is not guaranteed or endorsed by the publisher.
